# Propolis Use in Dentistry: A Narrative Review of Its Preventive and Therapeutic Applications

**DOI:** 10.1016/j.identj.2024.01.018

**Published:** 2024-02-19

**Authors:** Hayat Alghutaimel, Manal Matoug-Elwerfelli, Mayada Alhaji, Fatimah Albawardi, Venkateshbabu Nagendrababu, Paul Michael Howell Dummer

**Affiliations:** a College of Dentistry, King Saud bin Abdulaziz University for Health Sciences, Riyadh, Saudi Arabia; b King Abdullah International Medical Research Centre, Riyadh, Saudi Arabia; c Ministry of the National Guard–Health Affairs, Riyadh, Saudi Arabia; d College of Dental Medicine, QU Health, Qatar University, Doha, Qatar; e Department of Preventive and Restorative Dentistry, University of Sharjah, Sharjah, UAE; f School of Dentistry, College of Biomedical and Life Sciences, Cardiff University, Cardiff, UK

**Keywords:** Propolis, Bee glue, Flavonoids, Dentistry

## Abstract

Propolis is a resinous substance produced naturally by bees, and it consists of the exudates of plants mixed with enzymes, wax, and pollen. Propolis continues to gain considerable scientific interest due to its potential health benefits. The modern-day use of propolis in pharmaceutical preparations, such as toothpastes, mouthwashes, chewable tablets, mucoadhesive gels, and sprays, is increasing. However, the effectiveness of using propolis-containing pharmaceuticals in dentistry is not clear. The present paper aims to review the literature on the dental applications of propolis in preventive dentistry, periodontics, oral medicine, and restorative dentistry and discuss its clinical effectiveness. A literature search was conducted using Scopus, PubMed, and Web of Science databases. In total, 104 studies were included, of which 46 were laboratory studies, 5 animal studies, and 53 human clinical studies. Overall, the laboratory studies revealed a range of antimicrobial effects of propolis on oral pathogens. Clinical investigations of propolis in biofilm and dental caries control as well as adjuvant periodontal therapies reported positive outcomes in terms of plaque control, pathogenic microbial count reduction, and periodontal tissue inflammation control. Additional investigations included the use of propolis for the management of recurrent aphthous stomatitis, oral mucositis, and cavity disinfection after caries removal as well as the development of a range of restorative dental materials. Based on the reported outcomes of the studies, the clinical usage of propolis has potential. However, the majority of the evidence is derived from studies with flaws in their methodological design, making their results and conclusions questionable. As a consequence, properly designed and well-reported clinical studies are required to affirm the effectiveness of propolis for dental applications. Additionally, the safety of propolis and the optimal concentrations and extraction methods for its clinical use warrant further investigation. Utilisation of standardised propolis extracts will help in quality control of propolis-based products and lead to the achievement of reproducible outcomes in research studies.

## Introduction

Propolis is a resinous substance produced naturally by bees, and it consists of the exudates of plants mixed with enzymes, wax, and pollen. The term “propolis” is Greek in origin (from *pro* “in defense” and *polis* “the city”) and, in the context of bees, means “defense of the hive.” As the term suggests, propolis is used by bees for hive protection against bacteria, viruses, fungi, and parasites.^1^ It is also used to seal cracks and maintain temperature, humidity, and an aseptic environment within the beehive.^2^

The chemical composition and biological properties of propolis have been the subject of extensive research.^3^, ^4^, ^5^ These characteristics vary not only as a consequence of variations in the botanical sources of the constituents but also as a result of changes in the environmental conditions, making standardisation and quality control challenging. Although a range of constituents have been reported, crude propolis is generally composed of 50% plant resin and balsam, 30% beeswax, 10% essential and aromatic oils, 5% pollen, and 5% organic and inorganic compounds.^6^ The predominant chemical compounds of propolis are flavonoids, phenols (e.g. phenolic glycerides, phenolic acids and their esters, lignans, and stilbenes), terpenes, and terpenoids, with variable amounts of fatty acids, alcohols, alkaloids, sugar, amino acids, vitamins (e.g. B_1_, B_2_, B_3_, B_5_, B_6_, C, and E), and minerals (e.g. calcium, iron, potassium, magnesium, sodium, aluminium, boron, barium, chromium, manganese, nickel, strontium, and zinc).^1^ Propolis has a characteristic smell, and its colour varies from yellow to green and red to dark brown depending on the plant source and the collection season.^7^

The composition and biological properties of propolis are affected by the solvents and techniques used for its extraction. Ethanol, water, and propylene glycol have been used as solvents, and the selection of the most appropriate chemical depends on the intended use of propolis.^8^ In addition to the use of solvents, several propolis extraction techniques are used, including maceration and ultrasound-assisted, microwave-assisted, and supercritical carbon dioxide extraction.^1^ Amongst these techniques, ultrasound-assisted propolis extraction has been reported to be the optimal method in terms of extraction time and yield.^9^ The main source of commercial propolis is the honeybee, *Apis mellifera*, with less common types of propolis produced by stingless bee species, such as *Trigona sirindhornae*.^2^

The reported therapeutic and health-promoting effects of propolis include antibacterial,^10^^,^^11^ antiviral,^12^ antifungal,^13^ anti-inflammatory,^14^ antitumour,^15^ wound healing,^16^ and immunomodulation.^17^ The mechanism of action of propolis, although not well studied, has been described in the literature through its potential to activate the host natural defense and to impair the mobility, adherence, and metabolisms of microorganisms.^10^ For centuries, propolis was recognised as a therapeutic agent by several ancient cultures including the Egyptians and Greeks.^18^ However, its modern-day use in pharmaceutical preparations is increasing as its health benefits become more evident.^2^ Propolis-containing oral capsules, chewable tablets, lozenges, drops, sprays, creams, ointments, mucoadhesive gels, oral syrups, toothpastes, and mouthwashes are commercially available products, yet their clinical effectiveness is not clear.

In the medical field, the effectiveness of propolis has been investigated with variable success for the management of various infections and diseases, such as diabetic foot ulcers,^19^^,^^20^ skin wounds,^21^ cutaneous warts,^22^ respiratory tract infections,^23^, ^24^, ^25^ vaginal infections,^26^ muscle fatigue,^27^ and hypertension.^28^ Propolis use in the management of postoperative inflammation after excisional hemorrhoidectomy,^29^, ^30^, ^31^ inflammation and infection in patients undergoing hemodialysis,^32^, ^33^, ^34^ and inflammation in people with HIV infection has also been reported.^35^

Furthermore, there is growing interest within the dental field in the use of natural medicinal products with inherent antimicrobial activity, such as propolis, as alternative antimicrobials since antimicrobial resistance within individuals has become a serious global health threat.^36^ The antimicrobial efficacy of propolis against oral pathogenic microorganisms has been extensively assessed, with an overall strong efficacy reported.^37^, ^38^, ^39^, ^40^ Propolis not only represents a promising alternative antimicrobial agent but also has a range of favourable biological activities,^41^ making its use in preventing or treating various dental and oral conditions highly attractive. This narrative review aims to provide an overview on the applications of propolis in the fields of preventive dentistry, periodontics, oral medicine, and restorative dentistry, highlighting its clinical effectiveness and identifying research gaps that need to be addressed.

## Methods

An electronic literature search was conducted using Scopus, PubMed, and Web of Science databases up to and including September 2023. A combination of key words, including “propolis,” “bee glue,” “flavonoids,” “dental,” and “antimicrobial” were used in the databases following their syntax rules. All combinations using (AND, OR) were utilised to refine the search results. Primary studies focussed on propolis applications in preventive dentistry, periodontics, oral medicine, and restorative dentistry were included. Laboratory, animal, and human clinical studies were all included to provide a general comprehensive overview of evidence on the topic. No time restrictions were applied and only English records were included. Reviews, editorials, and conference proceedings were excluded. An additional manual search was carried out through reference mining of the identified studies.

All records were initially screened for relevance by 2 independent authors (MA and FA). Full texts of relevant papers were then retrieved and reviewed by 2 independent authors (HA and MME). Relevant data were extracted from the included studies and summarised in a narrative format and tables.

## Results and discussion

The electronic databases searche identified a total of 351 records, of which 100 duplicate records were removed and 166 records were excluded following initial title and abstract screening due to the lack of relevance to the scope of the present review. An additional 19 records were manually detected in the reference list of the included studies. In total, 104 studies were included in this review, of which 46 were laboratory studies, 5 were animal studies, and 53 were human clinical studies. Overall, the studies investigated the effectiveness of propolis usage in biofilm control, dental caries prevention, adjuvant periodontal therapy, the management of recurrent aphthous stomatitis, chemotherapy- and radiotherapy-induced oral mucositis, halitosis, denture stomatitis, cavity disinfection after carious tissue removal, and optimisation of the properties of restorative dental materials. The usage of propolis in various dental applications and delivery formulations (vehicles) is discussed below and summarised in the Figure.

**Fig:**
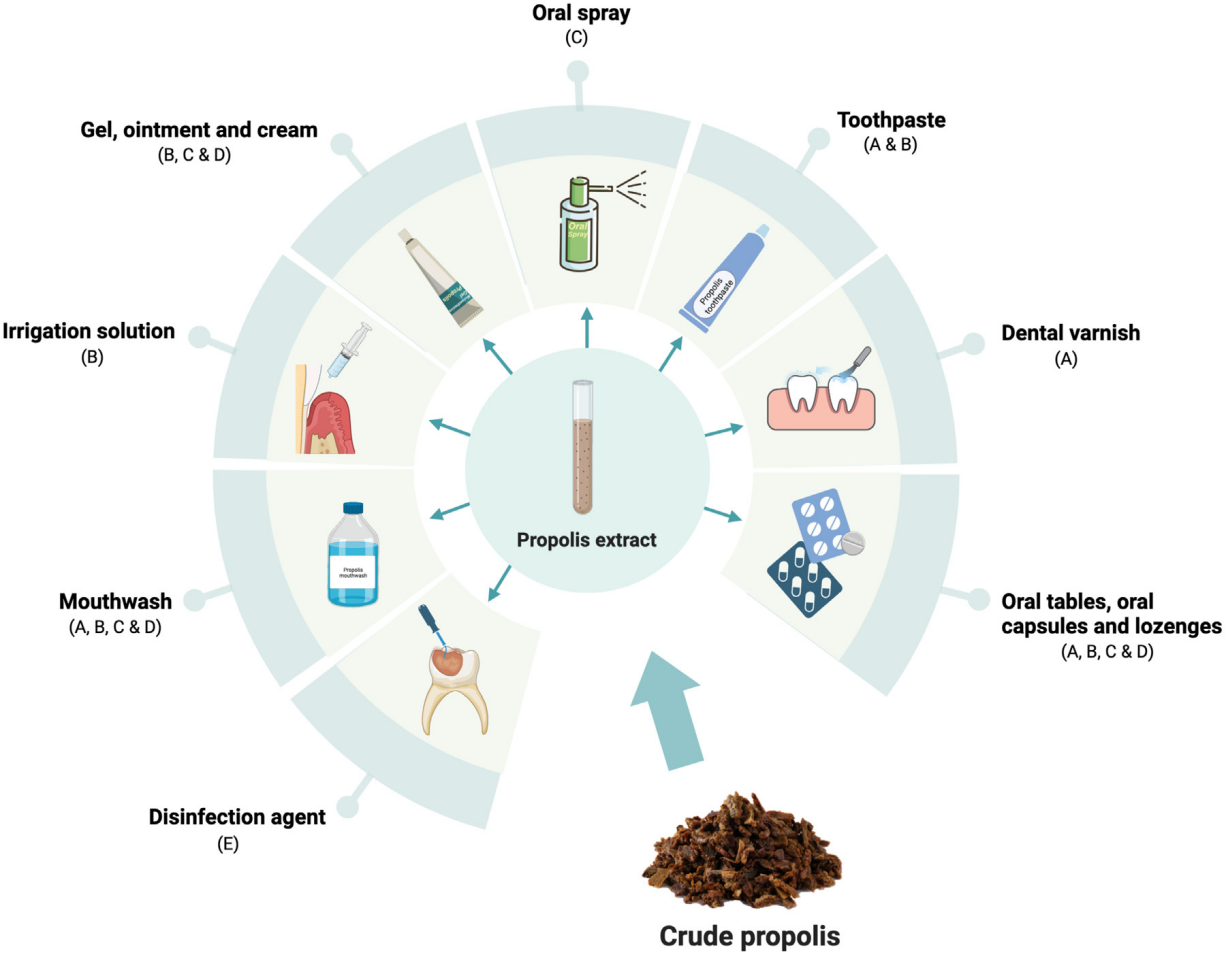
Schematic illustration of the investigated applications of propolis in preventive dentistry, periodontics, oral medicine, and restorative dentistry. A, Biofilm control and dental caries prevention; B, adjuvant periodontal therapy; C, Recurrent aphthous stomatitis; D, Chemotherapy- and radiotherapy-induced oral mucositis; E, Cavity disinfection after carious tissue removal

## Preventive dental applications

The effectiveness of propolis usage in preventive dentistry as an agent for biofilm control and dental caries prevention has been evaluated in laboratory, animal, and human clinical studies. A summary of the studies is discussed below, with further details of the human clinical studies, in terms of design, participants, groups, and reported outcomes, presented in Table 1.

**Table 1:** Human clinical studies on the effectiveness of propolis usage in biofilm and dental caries control.

Author (year)	Study design	Sample size	Age of participants (y)	Study group (n)	Control groups (n)	Propolis origin and extraction method (concentration)	Assessment method	Main findings
**Propolis-containing mouthwash**								
Bapat et al (2021)^69^	RCT	120	18–22	1-Hot ethanolic propolis extract mouthwash twice daily (30) 2-Cold ethanolic propolis extract mouthwash twice daily (30) 3-CHX mouthwash twice daily (30)	Distilled water twice daily for 3 mo (30)	Brazil propolis extracted using hot and cold ethanolic extraction methods (5 μg/mL)	-Plaque index was recorded at baseline, 15 d, 1 mo, and 3 mo. -Saliva was collected for microbiological analysis of *S mutans* at baseline, 5 min, and 1 h.	Propolis was found to be as effective as CHX in controlling plaque and reducing *S mutans* count.
Pereira et al (2011)^84^	Not clearly reported	25	18–60	Propolis-containing mouthwash for 1 min twice daily after brushing	No control	Brazil green propolis (5%) extracted using a solution containing glycerin, sodium benzoate, and water	Plaque and gingival index were assessed after 45 and 90 d of mouthwash use.	Significant reductions in plaque and in gingival index were observed.
Duailibe et al (2007)^72^	Not clearly reported	41	11–30	Propolis-containing mouthwash thrice daily (41)	No control	Propolis produced by stingless bee species, *Melipona compressipes fasciculata* (concentration was not reported)	Saliva samples were collected for microbiological analysis of *S mutans* at baseline and after 1 h and 7 d.	Reduction in the *S mutans* colony counts was observed in 49% of samples collected.
Koo et al (2002)^67^	RCT	6	20–38	Propolis-containing mouthwash (6)	Mouthwash without propolis (6)	Brazil propolis extracted using a combination of ethanol, propylene glycol, and deionised water (3%)	-Plaque index was recorded at day 4 of each experimental run. -Dental plaque was collected at day 4 of each experimental run for insoluble polysaccharides analysis.	Plaque index and insoluble polysaccharides were significantly reduced after the usage of propolis-containing mouthwash.
Murray et al (1997)^123^	RCT	42	Not reported	Propolis-containing mouthwash twice daily (13)	-CHX mouthwash twice daily (14) -Placebo mouthwash twice daily (14)	England propolis extracted using a combination of ethanol and water (10%)	Plaque score was recorded at baseline and after 5 d.	CHX mouthwash was found superior to propolis-containing mouthwash.
**Propolis-containing toothpaste**								
Lotif et al (2022)^74^	RCT	42	12–18	Fluoridated propolis-containing toothpaste (21)	Regular fluoridated toothpaste (21)	Brazil red propolis extracted using ethyl acetate (1%)	-Plaque index was recorded at baseline and after 4 wk. -Saliva was collected for microbiological analysis of *Lactobacillus* species.	-A significant reduction in plaque deposits was observed in both groups. -A significant reduction in the count of *Lactobacillus* species was observed in the study group.
Mohsin et al (2015)^85^	Not clearly reported	30	7–12	Propolis-containing toothpaste once daily for 4 wk (30)	No control	Commercially available toothpaste containing Korea propolis (Seoul Propolis Co.)	Plaque and saliva samples were collected for microbiological analysis at baseline and after 1, 3, and 4 wk.	Propolis toothpaste significantly reduced *S mutans* colony counts in the samples.
**Propolis-containing chewing gums and chewable tablets**								
Siqueira et al (2021)^81^	RCT	12	Not reported	Propolis- and xylitol-containing chewable tablets (6)	Placebo tablets without xylitol or propolis (6)	Brazil red propolis extracted using ethanol (concentration was not reported)	-Saliva samples were collected for microbiological analysis at baseline and immediately after chewing the tablet for 3–5 min. -Adverse reaction including itching, irritation, burning sensation, nausea, and laryngeal discomfort were also recorded.	-Propolis- and xylitol-containing chewable tablets resulted in a significant reduction of *S mutans* and Gram-negative bacteria colony counts in the saliva. -No signs/symptoms of adverse reactions were reported.
Martins et al (2020)^70^	RCT	27	10–19	Xylitol tablets (84%) with propolis (27)	Xylitol tablets (84%) without propolis (27)	Brazil red propolis extracted using ethanol (0.8%)	-Saliva samples were collected for microbiological analysis of *S mutans* at baseline and immediately after consumption of the tablets and after 7 d. -Dental biofilms were collected for microbiological analysis at baseline and after 7 d. -Participants’ feedback in terms of appearance, taste, aroma, and texture were recorded.	There was no difference between the effects of the tablets types on salivary flow, pH, and buffering capacity.
El-Allaky et al (2020)^79^	RCT	60	6–8	1- Propolis chewing gum twice daily for at least 20 min for 2 wk (30) 2- Propolis mouthwash twice daily for 1 min for 2 wk (30)	No control	Propolis extracted using propylene glycol and water (2%; propolis origin was not reported)	-Plaque index was recorded and a plaque sample was collected for microbial count at baseline and after 2 wk of treatment. -All participants were asked to rate the mouthwash they received during treatment period on a Visual Analogue Scale chart.	-Both formulations had a significant effect on plaque scores and colony counts with no differences between them. -Children preferred the gum formulation.
Tulsani et al (2014)^82^	RCT	30	8–11	Commercially available propolis chewing gum (15)	Commercially available xylitol chewing gum (15)	Commercially available France propolis (6.4%)	-Saliva samples were collected for microbiological analysis of *S mutans* at baseline and after 15 and 60 min of gum chewing. -Children's feedback in terms of taste acceptability was recorded.	-Propolis gum showed a statistically significant reduction in the number of *S mutans* colonies as compared to xylitol. -Xylitol gum was more preferred by the children.
**Propolis-containing dental varnish**								
Anani et al (2023)^78^	RCT	16 (patients with a total of 64 carious molars)	18–50	1- Varnish containing propolis (16) 2- Varnish containing hesperidin (16) 3- Varnish containing SDF (16)	No treatment (16)	Commercially available propolis (concentration and manufacturer were not reported)	-Radiographs were taken to assess remineralisation at baseline and after 12 wk. -Samples were collected from the cavities for microbiological analysis of *S mutans* at baseline and after 6 and 12 wk.	-The highest value of radiodensity was observed in the propolis group, whilst the lowest value was noted in the hesperidin group. -After 6 wk, the SDF group had the greatest reduction in bacterial counts, followed by the propolis group.
Neto et al (2021)^87^	RCT	75	3–5	-Varnish containing 2.5% propolis (25)	-Varnish containing 5% fluoride (25) -Varnish containing 1% CHX (25)	Brazil red propolis extracted using ethanol (2.5%)	Saliva samples were collected for microbiological analysis at baseline and after 3 and 6 mo.	Propolis-containing varnish was as effective as 5% fluoride varnish and 1% CHX varnish in reducing the *S mutans* colonies in saliva.
Neto et al (2020)^83^	RCT	24	3–5	1- Varnish containing 1% propolis (6) 2- Varnish containing 2.5% propolis (6) 3- Varnish containing 5% propolis (6) 4- Varnish containing 10% propolis (6)	No control	Brazil red propolis extracted using ethanol (1%, 2.5%, 5%, and 10%)	Stimulated saliva samples were collected for microbiological analysis before and after varnish application.	Microbiological reduction of *S mutans* was observed in all the tested concentrations with no significant differences between tested groups.

CHX, chlorhexidine; RCT, randomised clinical trial; SDF, silver diamine fluoride; d, day; h, hour; min, minute; mo, month; wk, week; y, year

### Laboratory studies

The antibiofilm effects of propolis on oral pathogenic microorganisms have been assessed in vitro for its inhibition of cellular attachment, growth, and metabolism as well as inhibition of glucosyltransferases enzymes production, which are essential for biofilm formation.^37^, ^38^, ^39^, ^40^^,^^42^, ^43^, ^44^, ^45^, ^46^, ^47^, ^48^, ^49^, ^50^, ^51^, ^52^, ^53^, ^54^, ^55^, ^56^, ^57^, ^58^, ^59^, ^60^, ^61^, ^62^, ^63^ Koo et al^45^ reported inhibition of microbial cell attachment and water-insoluble glucan formation by glucosyltransferases in cultures of *C albicans, S aureus, E faecalis, P gingivalis*, and *Streptococcus* species after exposure to variable concentrations of the ethanolic extract of Brazil propolis. The same group evaluated in vitro the antibiofilm effects of distinct chemical components found in Brazil propolis on various species of *Streptococcus* and reported apigenin and tt-farnesol as the most effective inhibitors of glucosyltransferases activity and microbial growth, respectively.^51^^,^^64^ Similar results were reported by others, revealing the antiadhesion effect of propolis on *S mutans* with inhibition of glucosyltransferases expression.^49^^,^^50^

Furthermore, several studies reported that the exact degree of propolis antimicrobial and antibiofilm efficacy is dependent on various factors, including source of origin, concentration, extraction solvent, length of exposure to the microorganisms, and type of microbial strain.^42^^,^^46^^,^^50^^,^^53^^,^^54^^,^^60^ Koru et al^56^ compared the antimicrobial activity of propolis extracts from 5 different regions of Turkey and Brazil against 9 strains of pathogenic oral anaerobic bacteria. The group reported susceptibility of all strains to propolis extracts at different concentrations and length of exposure, with inhibition of Gram-positive bacteria requiring lower concentrations compared to Gram-negative bacteria. Similar results have been reported by other research groups.^47^^,^^48^^,^^57^ Furthermore, several studies demonstrated variation on the microbial inhibitory concentrations of propolis extracted using different solvents, including ethanol, water, ether, acetone, methyl chloride, and benzene, suggesting a clear effect of the solvent on the antimicrobial activity of propolis.^58^^,^^65^

### Animal studies

Several animal studies investigated the effectiveness of propolis extracted using different solvents, including ethanol and hexane, as well as the effectiveness of propolis fractions, including apigenin and tt-farnesol, in reducing the incidence of dental caries in rats.^61^^,^^62^^,^^64^^,^^66^ The experimental model used in the studies involved Wistar rats infected with *Streptococcus sobrinus* and fed a cariogenic diet. Ikeno et al^61^ reported an approximate 60% reduction of fissure caries in rats exposed to ethanolic extracts of China propolis (1 mg/mL) for 48 days. However, the authors did not report the frequency of propolis extract administration. Koo et al^64^ treated rats’ teeth topically twice daily for 5 weeks with various materials, including (1) apigenin (0.035%, Extrasynthese), (2) tt-farnesol (0.028%, Sigma Aldrich), (3) a combination of apigenin and tt-farnesol, (4) sodium fluoride (0.05%), and (5) chlorhexidine gluconate (CHX, 0.12%). The authors reported reduction of dental caries incidence in rats exposed to a combination of apigenin and tt-farnesol, sodium fluoride, or CHX by 60%, 70%, and 72%, respectively, with no statistically significant differences amongst the 3 groups.^64^ Furthermore, Hayacibara et al^62^ and Arslan et al^66^ reported equal effectiveness of ethanolic and hexane extracts of Brazil propolis in dental caries reduction in rats after twice-daily topical application for 5 weeks.

### Human clinical studies

Numerous human observational studies and randomised clinical trials (RCTs) investigated the effectiveness of various formulations of propolis for use in biofilm and dental caries control, including propolis-containing mouthwashes,^67^, ^68^, ^69^, ^70^, ^71^ toothpastes,^72^, ^73^, ^74^, ^75^, ^76^, ^77^, ^78^ chewing gum and chewable tablets,^79^, ^80^, ^81^, ^82^ and dental varnishes.^83^, ^84^, ^85^, ^86^ A summary of the key findings is presented below.

### Propolis-containing mouthwash

The clinical effectiveness of propolis-containing mouthwashes in biofilm control was evaluated in several studies. In a preliminary RCT conducted by Koo et al,^67^ the use of a mouthwash containing 3% Brazil propolis extract for 1 minute twice daily for 3 days, as a solo oral hygiene measure, resulted in a statistically significant reduction in plaque deposits and insoluble polysaccharides content of the biofilm amongst healthy adult participants. However, as blinding of the outcome's assessor was not reported, the study possesses a risk of bias. In another observational clinical study, by Pereira et al,^84^ a statistically significant reduction in plaque deposits was reported amongst adult participants with inadequate oral hygiene who used a mouthwash containing 5% Brazil propolis extract for 1 minute twice daily after tooth-brushing for up to 3 months. However, the utilised study design has a risk of bias arising from lack of control over covariates and lack of randomisation. Furthermore, although Duailibe et al^72^ reported a reduction in *S mutans* colony counts in saliva samples of around half of participants after 7 days of thrice-daily rinsing with a mouthwash containing propolis extract, their study was not controlled. Methodical flaws were also noted in the study of Bapat et al,^69^ who reported comparable effectiveness of 0.2% CHX and a mouthwash containing 0.1% propolis from India in reducing *S mutans* and *L acidophilus* colony counts in the saliva of participants after twice-daily use for up to 3 months.

### Propolis-containing toothpaste

The benefit of using propolis-containing toothpaste has been assessed in several studies. In a clinical study by Mohsin et al,^85^ a statistically significant reduction in *S mutans* colony counts in dental plaque and saliva samples of children was reported after using commercially available toothpaste containing propolis from South Korea (Probee, Seoul Propolis Co.) once daily for 4 weeks. However, the study was not controlled and the results are likely to be biased. A recent double-blinded RCT by Lotif et al^74^ reported a statistically significant reduction in plaque deposits and *Lactobacillus* species count in saliva amongst adolescents with fixed orthodontic appliances and inadequate plaque control after twice-daily use of a toothpaste containing 1% propolis extract from Brazil for 28 days.

### Propolis-containing chewing gum and chewable tablets

Propolis-containing chewable gum and tablets have also been evaluated in several clinical studies, with variable reported outcomes. Tulsani et al^82^ reported a statistically significant reduction in *S mutans* colony counts in children's saliva after using chewing gum containing 6.4% propolis from France for 15 and 60 minutes. This is in agreement with El-Allaky et al,^79^ who reported a clear reduction in plaque deposits and biofilm microbial colony counts amongst children with high caries risk after using chewing gum containing 2% propolis from Egypt twice daily for 2 weeks. Although Martins et al^70^ reported that propolis did not enhance the properties of xylitol-containing chewable tablets, Siqueira et al^81^ demonstrated a statistically significant reduction in *S mutans* and Gram-negative bacteria colony counts in saliva samples of participants exposed to propolis and xylitol chewable tablets. This could be due to variations in the concentrations of active agents or frequencies of tablet consumption. Either way, further studies are needed, as all studies mentioned above have methodological design limitations and risk of bias.

### Propolis-containing dental varnish

The effectiveness of propolis-containing dental varnish in children with high caries risk was evaluated and compared to 5% fluoride varnish. Neto et al^83^ reported a significant reduction of *S mutans* colony counts in saliva samples of 3- to 5-year-old children following application of dental varnish containing as low as 1% propolis extract. Furthermore, Neto et al^87^ reported that 2.5% propolis varnish was as effective as 5% sodium fluoride varnish in reducing *S mutans* colony counts in participants’ saliva.

In summary, human clinical studies reported promising outcomes in terms of propolis usage as an agent for biofilm control. However, most of the studies have methodological flaws and bias arising from inadequate information on the randomisation process, lack of controls, lack of blinding, or missing or selective reporting of the outcome data, making clear conclusions impossible to draw. Therefore, further properly designed and well-reported clinical studies are required to confirm the caries-preventive effects of propolis. Additionally, further investigations are needed to reveal any side effects associated with the clinical applications of propolis and to determine its optimal concentration and extraction method. The utilisation of standardised propolis extract is essential to help in quality control of the products and achievement of reproducible outcomes.

## Periodontal applications

Propolis utilisation as an agent for adjuvant periodontal therapy has attracted scientific interest, with several laboratory and human clinical studies investigating its effectiveness in reducing pathogenic microbial counts, controlling tissue inflammation, and improving the overall health of periodontal tissues. A summary of the studies is provided below, with further details of the human clinical studies in terms of design, participants, groups, and reported outcomes presented in Table 2.

**Table 2:** Human clinical studies on the effectiveness of propolis usage in adjuvant periodontal therapy.

Author (year)	Study design	Sample size	Age of participants (y)	Study group (n)	Control groups (n)	Propolis origin and extraction method (concentration)	Assessment method	Main findings
**Propolis-containing mouthwash**								
Kiani et al (2022)^88^	RCT	32 participants with gingivitis	19–55	Mouthwash containing propolis extract (16)	Placebo mouthwash without propolis (16)	Commercially available propolis mouthwash (Impident, Gyahan Sabz Zendegy Co.)	BOP and teeth discolouration were evaluated at baseline and at 15 and 30 d.	-The reduction in BOP was significantly greater in the study group. -No teeth discolouration was noted in the study group.
Lisbona-González et al (2021)^89^	RCT	40 participants with chronic periodontitis and deep pockets	50–60	1- 2% propolis mouthwash thrice daily for 2 d (10) 2- 0.2% CHX mouthwash thrice daily for 2 d (10) 3- Propolis + CHX mouthwash thrice daily for 2 d (10)	Placebo mouthwash thrice daily for 2 d (10)	Spain propolis extracted using ethanol (10%)	Saliva samples were collected for microbiological analysis of *S mutans* and *Lactobacillus* at baseline, on the day the intervention started, and after 2 d.	Propolis mouthwash reduced *S mutans* and *Lactobacilli* colony counts, showing synergistic effect when combined with CHX.
Samad et al (2017)^77^	Not clearly reported	28	Not reported	1- Mouthwash containing 5% propolis (9) 2- Mouthwash containing 10% propolis (10)	Placebo mouthwash (9)	Propolis produced by stingless bee species, *Trigona sirindhornae* (5% and 2%)	Microbiological evaluation for Gram-negative anaerobic bacteria in gingival crevicular fluid at baseline and after 14 d.	A statistically significant reduction in anaerobic bacterial colony count was observed in the study groups.
**Propolis-containing toothpaste**								
Machorowska-Pieniążek et al (2021)^90^	RCT	50 participants with oral clefts and orthodontic appliances	9–16	Propolis toothpaste (25)	Placebo toothpaste (25)	Poland propolis extracted using ethanol (1.5%)	-PD and BOP were recorded at baseline and after 35 d.	-A decrease in PD and BOP were observed in the study group.
Hassan and Ahmed (2021)^86^	RCT	20 participants with gingivitis	20–35	1- Propolis -containing toothpaste (20) 2- Commercially available toothpaste (Colgate Palmolive Co.) (20)	No control	Commercially available propolis toothpaste (Ecodenta triple force, BIOK Laboratory)	-PD and BOP were recorded at baseline and after 7 d. -Analysis of interleukin-1β and interleukin-6 levels in saliva at baseline and after 7 d.	-A statistically significant reduction in PD, BOP, and interleukin levels were reported in the study group.
Lisbona-González et al (2021)^89^	RCT	60 participants with chronic periodontitis and teeth extraction	50–60	Propolis paste 3 times a day for 1 wk (30)	Placebo paste 3 times a day for 1 wk (30)	Spain propolis extracted using ethanol (10%)	-Visual examination of socket healing was performed on the 3rd, 4th, and 7th day.	-Complete healing was observed in 90% of the propolis group compared to 13.4% in the control group.
Peycheva et al (2019)^80^	RCT	70 participants with gingivitis	12–18	Toothpaste with 10 drops of propolis extract (35)	Toothpaste without propolis (35)	Bulgaria propolis extracted using hydro-alcohol (20%)	-PD and BOP were recorded at baseline and after 20 d. -Dental plaque samples were collected for microbiological analysis at baseline and after 20 d.	-A statistically significant reduction in PD and BOP was seen in both groups before and after the product's usage. -A statistically significant reduction of BOP was seen in the study group compared to the control group. -Eradication of *S mutans, C albicans, F varium*, Gram-negative cocci, Gram-positive rods, *P bivia, P intermedia, P melani*, and *S intermedius* was observed in the study group.
Skaba et al (2013)^73^	Not clearly reported	32 participants with the following characteristics: -Group 1: with obvious plaque deposits and gingivitis (16) -Group 2: without obvious plaque deposits or gingivitis (14)	Not reported	Toothpaste with propolis Group 1: (9) Group 2: (7)	Toothpaste without propolis Group 1: (9) Group 2: (7)	Brazil green propolis extracted using ethanol (3%)	-PD and BOP were recorded at baseline and after 1 and 4 wk. -Microbiological analysis of *S mutans* in saliva samples were performed at baseline and after 4 wk.	-PD and BOP in the study groups improved significantly. -*S mutans* colony counts in saliva were reduced.
Morawiec et al (2013)^75^	Not clearly reported	16 participants with implant supported prosthesis	22–65	Toothpaste with propolis (8)	Toothpaste without propolis (8)	Brazil red propolis extracted using ethanol (3%)	-PD, BOP, and PPD were recorded at baseline and after 1 and 8 wk. -A questionnaire was used to assess patients' opinions on the rheological and organoleptic properties of the propolis-containing toothpaste.	-A statistically significant reduction in PD and BOP was observed in the study group. -The main concern with propolis toothpaste was the colour.
**Propolis-containing oral capsules**								
Park et al (2021)^91^	RCT	104 participants diagnosed with gingivitis or incipient periodontitis and having at least 1 tooth with pocket of >3 mm and ≤5 mm	20–75	Daily intake of a single capsule containing 194 mg of mangosteen and propolis for 8 wk (52)	Daily intake of a single placebo capsule for 8 wk (52)	Not reported	-PD, BOP, and PPD were recorded at baseline and after 4 and 8 wk. -Immunologic analysis of saliva and gingival crevicular fluid samples at baseline and after 4 and 8 wk.	No statistically significant differences were found between the study and control group for all parameters tested.
El-Sharkawy et al (2016)^92^	RCT	50 participants with type 2 diabetes and moderate to severe chronic periodontitis	38–63	Propolis capsule orally, once daily for 6 mo + scaling and root planing (24)	Placebo + scaling and root planing (26)	Propolis capsules (400 mg; BioPropolis, Sigma Pharmaceutical Industries)	PD, BOP, and PPD were recorded at baseline and after 3 and 6 mo.	No statistically significant differences were found between the study and control groups in terms of reduction of PD, BOP, and PPD.
**Propolis-containing ointment and gel**								
González-Serrano et al (2023)^95^	RCT	22 participants with desquamative gingivitis	≥18	Gel containing propolis extract (11)	Placebo gel (11)	Propolis gel (2%; Sungwon Pharmaceutical Co.)	Clinical periodontal parameters on the extent and severity of the desquamative gingivitis lesions and Visual Analog Scale for pain and oral health impact profile were collected at baseline and at 2 and 4 wk.	Significant improvement of the clinical periodontal parameters was seen in the study group.
Nakao et al (2020)^93^	RCT	24 participants with moderate to advanced chronic periodontitis who completed initial periodontal therapy and have at least 1 tooth with periodontal pockets ≥5 mm	27–73	1- 10 mg/mL propolis in carboxymethyl cellulose sodium salt ointment (6) 2- 1 mg/mL curry leaf in carboxymethyl cellulose sodium salt ointment (5) 3- 2% minocycline hydrochloride ointment (6) Ointments were professionally applied in the pockets 3 times per month	Placebo of carboxymethyl cellulose sodium salt ointment (6)	Brazil propolis extracted using ethanol (1%)	-BOP, PPD, and CAL were recorded at baseline and after 3 mo. - Microbiological analysis of samples from the gingival crevicular fluid at baseline and after 3 mo.	Propolis usage improved PPD and CAL with a tendency towards reducing *P gingivalis* in the gingival crevicular fluid.
González-Serrano et al (2021)^96^	RCT	46 participants with peri-implant mucositis	Not reported	Gel containing propolis extract (23)	Placebo gel (23)	Korea propolis extracted using ethanol (2%)	-PD, BOP, and PPD were recorded at baseline and after 1 mo. -Microbiological analysis at baseline and after 1 mo.	-26.1% of participants in the study group showed complete resolution of BOP compared to none in the control group. -Significant reductions in PD and PPD were seen in the study group. -Reduction in the colony counts of *T forsythia* and *P gingivalis* were seen in the study group.
Giammarinaro et al (2018)^94^	Not clearly reported	40 participants	18–70	1- Gel containing propolis after initial periodontal therapy (20) 2- Gel containing CHX after initial periodontal therapy (20)	No control	Propolis gel (Oralsan NBF gel, IDS Spa)	-PD, BOP, and PPD were measured at baseline and after 1 and 3 mo. -Salivary antioxidant capacity was also measured.	-No statistically significant differences in PD, BOP, and PPD were seen between the groups. -The study group had better results in terms of oxidative stress reduction.
**Propolis-containing irrigation solution**								
Seth et al (2022)^97^	RCT	20 participants	18–55	1- Subgingival irrigation with propolis extract after scaling and root planing (10) 2- Subgingival irrigation with 0.2% CHX after scaling and root planing (10)	No control	Propolis extract (Super Bee Propolis tincture, Hi-Tech Natural Products India Ltd.) (25%)	-PD, BOP, and PPD were assessed at baseline and after 15 and 30 d. -Microbial analysis for the colony-forming unit was done at baseline and after 30 d.	-A statistically significant reduction was observed in PD, BOP, PPD, and colony-forming unit counts from baseline to 30 d in both groups. -Intergroup comparison revealed that the group receiving irrigation with CHX showed slightly better results.
Pundir et al (2017)^76^	Not clearly reported	30 participants with chronic periodontitis and presenting ≥3 nonadjacent teeth with deep pockets	25–55	20% propolis hydroalcoholic mouthwash after scaling and root planing (15)	Scaling and root planing alone (15)	India propolis extracted using ethanol (20%)	PD, BOP, and PPD were recorded followed by subgingival plaque sampling at baseline and after 4 and 12 wk.	A statistically significant improvement in PD, BOP, and PPD was seen in the study group.
Coutinho et al (2012)^124^	Not clearly reported	20 participants with chronic periodontitis who completed initial periodontal therapy and have a minimum of 3 nonadjacent teeth with deep pockets	25–57	Hydroalcoholic solution of propolis extract twice a week for 2 wk (10)	1- Placebo of 14% ethanol solution twice a week for 2 wk (5) 2- No additional treatment after ultrasonic scaling and root planing (5)	India propolis extracted using ethanol (20%)	-PD, BOP, PPD, and CAL were recorded at baseline and after 4, 6, and 8 wk. -Subgingival plaque samples were also collected for microbiological analysis.	-A statistically significant reduction in BOP was seen. -Reduction of *P gingivalis* and anaerobic bacterial counts was observed in the study group.

BOP, bleeding on probing; CAL, clinical attachment level; CHX, chlorhexidine; PD, plaque deposits; PPD, periodontal pocket depth; RCT, randomised clinical trial; d, day; h, hour; min, minute; mo, month; wk, week; y, year

### Laboratory studies

The effects of propolis extracts on various periodontal pathogens was assessed in vitro^52^^,^^54^^,^^59^^,^^62^ and compared to well-established antimicrobials, including amoxicillin,^46^ and CHX.^37^^,^^39^ It has been reported that both Brazil red propolis and amoxicillin exhibited similar reductions in colony counts and metabolic activity of multiple microorganisms associated with periodontal diseases after 24 hours of exposure.^55^ However, it is not clear whether the antimicrobial effectiveness of propolis will continue to be comparable to that of amoxicillin after shorter exposure times. Furthermore, although propolis extracts from Turkey and Brazil were reported to be as effective as CHX on biofilms of streptococci and fungi, CHX was more effective against biofilms of *A actinomycetemcomitans, S aureus*, and *E faecalis*.^37^^,^^39^

### Human clinical studies

#### Propolis-containing mouthwash

The addition of propolis, as a natural active agent, to mouthwashes has been assessed in human clinical studies. Kiani et al^88^ reported a statistically significant reduction in gingival sulcus bleeding amongst adult patients with gingivitis, who were otherwise healthy, after twice-daily use of propolis mouthwash for 2 weeks, with the conventional tooth-brushing and dental flossing. However, it is not clear whether this result was because of propolis mouthwash usage or merely adherence to regular tooth-brushing and dental-flossing regimens amongst the participants. Lisbona-González et al^89^ and Samad et al^77^ reported positive outcomes of propolis mouthwash usage in patients with periodontitis and deep periodontal pockets. However, both studies are likely to be biased due to inadequate control over covariates.

#### Propolis-containing toothpaste

The addition of propolis to toothpastes has been assessed in several human clinical studies. In a randomised controlled trial by Machorowska-Pieniążek et al,^90^ adolescents with cleft lip and/or palate, gingivitis, and orthodontic appliances were exposed to propolis toothpaste thrice daily for 35 days. Although the authors reported reduction of plaque deposits and gingival sulcus bleeding, such findings could occur due to adherence to conventional oral hygiene practice and not because of any additional therapeutic effect. Hassan and Ahmed^86^ reported reduction of gingival sulcus bleeding and inflammatory interleukin levels in the saliva of patients with gingivitis who used propolis toothpaste twice daily for 7 days. This is in agreement with the results reported by Peycheva et al,^80^ who reported reduction of gingival sulcus bleeding along with eradication of multiple periodontal pathogens following the use of toothpaste supplemented with drops of propolis extract twice daily for 1 and 4 weeks. However, the results of the previously mentioned studies, together with those of other similar studies,^73^^,^^75^ are likely to be biased due to inadequate control over the covariates.

#### Propolis-containing oral capsules

The production of oral capsules (to be swallowed) incorporated with propolis as an adjunct to periodontal therapy has been reported on a limited basis. In a multicentre RCT by Park et al,^91^ adult patients with gingivitis or incipient periodontitis were subjected to daily intake of a single oral capsule containing 194 mg mangosteen and propolis for 4 and 8 weeks, in addition to conventional oral hygiene practice. Although the authors reported no significant differences between the test and control groups in terms of plaque deposit reduction, bleeding on probing, periodontal pocket depth improvement, and change in inflammatory markers, the study had selection bias, as outcomes from patients with gingivitis cannot be compared to those from patients with periodontitis and deep pockets.^91^ El-Sharkawy et al^92^ reported absence of a clear effect of propolis oral capsules (400 mg) as adjuvants to scaling and root planing in patients with moderate to severe chronic periodontitis and type 2 diabetes mellitus.

#### Propolis-containing ointment and gel

Similar to the above reported findings, the production of ointments and gels with incorporated propolis as an adjunct to periodontal therapy to reduce plaque deposits has been explored. In a preliminary RCT by Nakao et al,^93^ adult patients with moderate to severe chronic periodontitis were exposed to professional application of propolis ointment (10 mg/mL) in the periodontal pockets thrice monthly after the initial periodontal therapy. Although the authors reported improvement of periodontal pocket depths and clinical attachment levels with reduction of *P gingivalis* colonies in the gingival crevicular fluid at 3 months,^93^ there is a risk of bias arising from lack of information about standardisation of oral hygiene practices amongst the participants over the study period. Giammarinaro et al^94^ reported equal effectiveness of topically applied propolis gel and CHX gel, in terms of resolution of gingival sulcus bleeding and improvement of periodontal pocket depths, in adult patients with chronic periodontitis who completed the initial periodontal therapy. However, the study has design limitations, and all recruited participants had gingivitis only with no pathologic periodontal pockets. Inadequate control over the covariates was also noted in the other studies reporting positive results in association with propolis ointments and gels as an adjunct to periodontal therapy.^95^^,^^96^

#### Propolis-containing irrigation solution

The use of propolis as an active agent in periodontal irrigation solutions has also been assessed. Pundir et al^76^ and Coutinho et al^124^ reported reductions in periodontal pocket depths and bleeding on probing following the use of propolis as a subgingival irrigation solution in patients with chronic periodontitis. However, Seth et al^97^ reported that 0.2% CHX was superior to 25% propolis extract as a subgingival irrigation solution after scaling and root planing in patients with chronic periodontitis and deep pockets.

In summary, although the clinical studies discussed here reported positive outcomes for propolis products used as adjuncts to periodontal therapy, valid scientific conclusions cannot be drawn due to methodological flaws in the reported studies. Therefore, further clinical studies that are properly designed and well conducted, with comprehensive and unbiased reporting of outcomes, are needed.

## Oral medicine applications

### Recurrent aphthous stomatitis (RAS)

#### Human clinical studies

Propolis use as a remedial therapeutic agent in idiopathic RAS, in otherwise healthy individuals, has attracted scientific interest owing to its anti-inflammatory, antioxidant, immunomodulatory, and wound-healing properties. Several propolis-based products have been tested clinically in humans, including mouthwash,^98^ solutions for topical application,^99^ spray,^100^^,^^101^ mucoadhesive film,^102^ paste,^103^ and oral capsules.^104^ The human clinical studies are discussed below, with further details, in terms of design, participants, groups, and reported outcomes, presented in Table 3.

**Table 3:** Human clinical studies on the effectiveness of propolis usage in recurrent aphthous stomatitis.

Author (year)	Study design	Sample size*	Age of participants (y)	Study group (n)	Control groups (n)	Propolis concentration/origin and extraction method	Assessment method/follow-up	Main findings
**Propolis-containing mouthwash**								
Tonkaboni et al (2016)^98^	RCT	45	18–60	Propolis mouthwash thrice daily for 3 mo (22)	Placebo mouthwash thrice daily for 3 mo (23)	3% propolis (Sorentech Company)	Size of ulcer, pain, and time until healing were assessed at baseline and after 3 mo.	A statistically significant difference between the groups and within each group, in terms of ulcer size reduction, pain intensity, and ulcer healing.
**Propolis-containing solution for topical application**								
Lotufo et al (2005)^99^	Prospective clinical study	40**	15–60	Topical application of propolis solution thrice daily (40)	No control	5% Brazil propolis in ethanol, propylene glycol, and deionised water	Time until healing and ulcer recurrence were assessed after 7 d and subsequently every 15 d for 1 y.	Reduction in time until healing and recurrence were observed.
**Propolis-containing spray**								
Rodríguez-Archilla and Raissouni (2017)^100^	RCT	125**	8–56	1- Cauterisation with silver-nitrate in the clinic (25) 2- Propolis spray thrice daily (25) 3- 5% Rhubarb extract thrice daily (25) 4- 5% Walnut extract thrice daily (25)	Placebo spray of flavoured distilled water thrice daily (25)	18% Oropropolis spray (Buccal, Laboratoire Pharmasoft)	Time to remission of symptoms and lesion healing were recorded through daily follow-up.	-All study groups performed better than the placebo group. -Propolis spray resulted in symptoms resolution after 1.6 d and complete healing after 6.8 d. -The fastest symptom resolution was noted in group 1, and the shortest healing time was in group 2.
Atanasovska Stojanovska et al (2014)^101^	RCT	20	20–30	Propolis spray thrice daily (10)	Placebo spray thrice daily (10)	25% Propolis in diluted alcohol (propolis origin was not reported)	Lesion size and pain intensity were recorded at baseline and after 3, 5, and 8 d.	-Propolis spray resulted in faster reduction in ulcer dimensions. -The magnitude of pain was also reduced in the study group.
**Propolis-containing mucoadhesive film**								
Arafa et al (2018)^102^	RCT	24	≥18	Propolis mucoadhesive film twice daily (12)	Placebo mucoadhesive film twice daily (12)	Ethanolic and water extract of Egypt propolis (3%)	Ulcer size reduction, time until healing, and pain relief were recorded by the participants using a structured checklist.	A statistically significant reduction of ulcer sizes, time until healing, and pain relief were observed in the study group.
**Propolis-containing paste**								
Ali and Abdul Rasool (2011)^103^	Not clearly reported	120	>19	1- Propolis paste with sesame oil twice daily (40) 2- Propolis paste with olive oil twice daily (40)	Placebo paste without propolis (40)	Alcoholic extract of propolis (20%)	Reduction in ulcer size, time until pain is relieved, and time until healing were recorded.	Both propolis paste formulas resulted in a statistically significant reduction in ulcer size and pain with less time until ulcer healing.
**Propolis-containing oral capsules**								
Samet et al (2007)^104^	RCT	19	≥18	500 mg propolis capsule daily (10)	500 mg placebo capsule of a calcium-based food supplement daily (9)	Commercially available propolis (Vitamin World, no further information reported about the manufacturer)	Frequency of ulcer recurrence over 13 mo was recorded through biweekly follow-up.	Propolis usage was found effective in reducing RAS recurrence rate in 50% of the participants.

RAS, recurrent aphthous stomatitis; RCT, randomised clinical trial; d, day; h, hour; min, minute; mo, month; wk, week, y, year

⁎ Participants with RAS.

⁎⁎ Study specifically mentioned participants with minor RAS.

The effectiveness of propolis mouthwashes amongst adult patients with minor RAS was assessed in one RCT, with promising results reported in terms of time until ulcer healing and pain control.^98^ Furthermore, improved healing was reported amongst adult patients with RAS following topical application of propolis-based solution 3 times a day.^99^ Accelerated ulcer epithelisation with reduction of ulcer dimension and pain intensity was also reported following usage of propolis spray 3 times a day amongst adult patients with minor RAS.^101^ Consistent results were reported in another study, demonstrating pain resolution and ulcer healing following 2 and 7 days, respectively, of propolis spray usage thrice daily in comparison to 4 days for pain resolution and 9 days for ulcer healing in the control group.^100^ However, the latter study reported an association between silver nitrate cauterisation and better symptom resolution in RAS cases. Further studies are needed to better understand the therapeutic potential of propolis in comparison to traditional agents for the management of RAS.

In addition to the above, the inclusion of propolis extract in mucoadhesive films was evaluated in preliminary clinical study involving 24 adult patients with RAS. The authors reported effective reduction of ulcer dimension, time until healing, and pain intensity following the usage of propolis-based mucoadhesive film twice daily.^102^ It was suggested that this delivery vehicle could be superior to other investigated vehicles due to the sustained release of propolis and targeted delivery with anti-infective barrier formation between the ulcer and the oral cavity. However, evidence supporting this hypothesis is missing.

In summary, promising clinical results have been reported in the literature regarding propolis usage to accelerate healing and improve quality of life amongst patients with RAS who are otherwise healthy. However, further studies are needed to address several methodological flaws in the reported studies, such as lack of blinding and control over covariates.

## Chemotherapy- and radiotherapy-induced oral mucositis

### Human clinical studies

The medicinal properties of propolis make it an attractive agent for the management of chemotherapy- and radiotherapy-induced oral mucositis.^105^ Several formulations and delivery vehicles have been investigated, including propolis-containing mouthwash,^106^, ^107^, ^108^, ^109^, ^110^ mucoadhesive gel,^111^ paste,^112^ and oral tablets.^113^ A summary of the studies is given below, with further details, in terms of design, participants, groups, and reported outcomes, presented in Table 4.

**Table 4:** Human clinical studies on the therapeutic effectiveness of propolis in chemotherapy and radiotherapy-induced oral mucositis.

Author (year)	Study design	Sample size	Age of participants (y)	Study group (n)	Control groups (n)	Propolis concentration/origin and extraction method	Assessment method/follow-up	Main findings/success rate
**Propolis-containing mouthwash**								
Hamzah et al (2022)^106^	RCT	17 participants undergoing radiotherapy for nasopharyngeal carcinoma	≥18	Propolis mouthwash 3 times a day for 6 wk (10)	Placebo mouthwash (7)	2.5% Propolis (no information reported about extraction method or manufacturer)	The severity of OM was evaluated using the WHO scale after 2, 4, and 6 wk.	A statistically significant reduction in OM severity score was observed in the study group.
Dastan et al (2020)^109^	RCT	30 participants undergoing radiotherapy for various types of head and neck cancers	≥18	Propolis mouthwash thrice daily for 4 wk (15)	Placebo mouthwash thrice daily for 4 wk (15)	0.8 mg/mL propolis (Soren Tektoos)	The severity of OM and dysphagia were evaluated using NCI-CTC and CTCAE at each weekly visit.	Statistically significant differences were observed between the groups in the 2nd, 3rd, and 4th week of radiotherapy.
Marucci et al (2017)^107^	RCT	104 participants undergoing chemoradiotherapy for head and neck cancer	≥18	Mouthwash containing propolis, aloe vera, calendula, and chamomile 4 times a day for 5 wk (52)	Placebo mouthwash (52)	6% Propolis (Faringel, Cadigroup)	The severity of OM was assessed using CTCAE at each weekly visit.	Propolis mouthwash was found to be ineffective in preventing severe OM.
Akhavankarbassi et al (2016)^108^	RCT	40 participants undergoing chemotherapy for head and neck cancer	≥18	Propolis mouthwash thrice daily for 7 d (20)	Placebo mouthwash (20)	30% Propolis (Soren Tektoos)	The severity of OM was evaluated using the WHO scale at baseline and after 3 and 7 d.	-Propolis mouthwash resulted in quick improvement of OM and participants’ ability to eat and drink. -65% of the patients in the study group showed complete OM healing at day 7.
Eslami et al. (2016)^110^	RCT	72 participants undergoing chemotherapy for leukaemia	≤18	1- Mouthwash of propolis, CHX, and fluconazole thrice daily for 14 d 2- A combination of artificial saliva spray and mouthwash of CHX and fluconazole	Mouthwash of CHX and fluconazole	Not reported	The severity of OM was evaluated using the WHO scale.	None of the patients in the propolis group had severe OM after 5 and 10 d of chemotherapy.
**Propolis-containing mucoadhesive gel**								
Noronha et al (2014)^111^	Not clearly reported	24 participants undergoing radiation therapy for head and neck cancer	38–72	Propolis mucoadhesive gel 3 times a day for 2 wk starting 1 d before the course of radiation therapy (24)	No control	Ethanolic extract of Brazil green propolis (5%)	Food intake and grading of OM using WHO scale was performed at each weekly visit.	-20 patients did not experience OM, 2 patients experienced WHO grade 1 OM, and 2 patients experienced WHO grade 2 OM. -None of the patients discontinued food intake.
**Propolis-containing paste**								
Tomaževič and Jazbec (2013)^112^	RCT	50 participants undergoing chemotherapy	1–19	Propolis paste twice daily (26)	Placebo paste twice daily (24)	Ethanolic extract of China propolis (70%)	OM severity was assessed twice a week using the Modified Eilers Oral Assessment scale.	Propolis had no effect on OM severity.
**Propolis-containing oral tablets**								
Piredda et al (2017)^113^	RCT	60 participants undergoing chemotherapy for breast cancer	≥20	Mouthwash with sodium bicarbonate followed by chewing and swallowing propolis tablet (80 mg) 2–3 times a day (30)	Mouthwash with sodium bicarbonate 2–3 times/day (30)	80 mg propolis tablets (Natur Farma, S.A.S, no further information reported about manufacturer)	OM severity was assessed using NCI-CTC at 5, 10, 15, and 21 d of the first chemotherapy cycle.	Reduction of severe OM incidence was observed in the study group.

CHX, chlorhexidine; CTCAE, Common Terminology Criteria for Adverse Events; OM, oral mucositis; NCI-CTC, National Cancer Institute Common Toxicity Criteria; WHO, World Health Organization; d, day; h, hour; min, minute; mo, month; wk, week; y, year

The effect of propolis mouthwash on the severity of oral mucositis amongst adult patients undergoing radiotherapy and chemotherapy for head and neck cancers has been assessed, with variable outcomes. Hamzah et al^106^ and Akhavankarbassi et al^108^ reported reductions in oral mucositis severity scores (assessed using the World Health Organization [WHO] scale) and improvement of participants’ ability to eat and drink following the use of propolis mouthwash thrice daily for up to 6 weeks starting from the date of therapy initiation.^106^^,^^108^ On the contrary, Marucci et al^107^ concluded that there was no effect of mouthwashes containing propolis, aloe vera, calendula, and chamomile on the severity of chemotherapy-induced oral mucositis following its use 4 times daily for 5 weeks. A potential explanation of the variation in the outcomes amongst the studies could be variations in cancer therapy protocol, the demographics of the participants, and the oral mucositis assessment scales used. Furthermore, outcome variation amongst the studies could also be due to differences in propolis concentrations, origin, and extraction methods.

In addition, variable outcomes were reported following the usage of propolis-containing mucoadhesive gels, paste, and oral tablets (to be swallowed) amongst adult and children undergoing chemotherapy and radiotherapy for various types of cancers. Noronha et al^111^ reported no incidence of severe oral mucositis (based on the WHO scale) following the use of propolis mucoadhesive gel thrice daily starting 1 day before radiotherapy amongst adults with head and neck cancer. Similarly, Piredda et al^113^ demonstrated the reduction in the incidence of severe oral mucositis (based on the National Cancer Institute Scale) following the use of propolis oral tablets and sodium bicarbonate mouthwashes amongst female patients undergoing chemotherapy for breast cancer. In contrast, Tomaževič and Jazbec^112^ reported no effect on oral mucositis severity (based on the modified Eilers oral assessment scale) following the use of propolis paste amongst children undergoing chemotherapy for unspecified types of cancer.

In summary, although researchers have uncovered positive results, suggesting the potential benefit of propolis in the management of chemotherapy- and radiotherapy-induced oral mucositis, further studies are needed utilising controlled designs, standardised assessment methods, and standardised propolis extracts in order to arrive at sound scientific conclusions.

## Miscellaneous oral medicine applications

Propolis usage has also been tested clinically for the management of denture stomatitis and morning halitosis. Improvement in denture stomatitis with reduction of *Candida* yeast was reported amongst a small sample of full denture wearers following twice-daily rinses with propolis-containing mouthwash for 2 weeks.^13^ Furthermore, a study reported a statistically significant reduction in the count of volatile sulfur compound–producing microorganisms amongst healthy adults with morning halitosis, following twice-daily rinsing with propolis-containing mouthwash for 5 days.^114^ Although promising clinical results were published in both studies, they were preliminary in nature and had inadequate control over several confounding factors. Therefore, further studies are needed to confirm the results.

## Restorative dental applications

### Laboratory studies

Propolis has attracted attention because of its potential benefits when incorporated into dental materials and used for cavity disinfection during dental caries removal. The development of a propolis-containing bioadhesive system for dental pulp protection and propolis-containing nanohydroxyapatite with antimicrobial activity against *S aureus* has been reported.^115^, ^116^, ^117^, ^118^ Furthermore, addition of propolis to glass ionomer cement in order to enhance its biological properties has been attempted, with variable success. Abdallah et al^119^ demonstrated, in a laboratory study, no impact on the colour stability and microhardness of ceramic-reinforced glass ionomer cement following the addition of 50% ethanolic extract of Egyptian propolis. On the contrary, Panahandeh et al^120^ concluded that addition of 25% and 50% aqueous extract of Iranian propolis to resin-modified glass ionomer cement reduced the flexural and shear bond strength of the material with no apparent in vitro antimicrobial activity against *S mutans*.

### Human clinical studies

Propolis use as a cavity disinfection agent following dental caries excavation has been investigated clinically on a limited basis.^121^ In an RCT by Prabhakar et al,^122^ reduction of microbial colony counts was reported following propolis applications for 1 minute after manual caries excavation in primary molars as part of the atraumatic restorative technique. However, due to the limited number of clinical studies, further research is needed to confirm the results.

## Limitations, concluding remarks, and future perspectives

The authors acknowledge the limitations of the current review, which are mainly associated with the relatively poor quality of the included studies. The majority of the included studies had methodological flaws and bias arising from either inadequate information on the randomisation process, lack of control over confounding factors, lack of blinding, and missing or selective reporting of the outcome data, making their conclusions questionable. Furthermore, the methodological variations amongst clinical studies make comparisons impossible. A strength of the study lies in the fact that the authors ensured that all data screening and extraction were performed by 2 independent and calibrated authors to ensure the quality and accuracy of the extracted data. To gain an overall view of propolis usage within the dental field, a narrative study design was adopted.

In summary, various propolis-based products are available to clinicians and the general public as over-the-counter medications. Although the available literature demonstrates overall promising clinical outcomes when propolis is used, future researchers are encouraged to standardise methodological designs, propolis extraction methods, and assessment methods in order for their results and conclusions to be valid and to be compared. Furthermore, to streamline commercial production, clear guidelines for quality control and assurance of this natural product should be developed. Studies of the individual constituents of propolis will help to better understand its mechanism of action and aid the process of quality control and standardisation.

## Conflict of interest

None disclosed.
